# Association between Anti-inflammatory Drug and Dementia in Patients with Gout: A Nationwide, Population-Based Nested Case-Control Study

**DOI:** 10.7150/ijms.55496

**Published:** 2021-03-10

**Authors:** Natalia Mikhailichenko, Yu-Hsun Wang, James Cheng-Chung Wei, Te-Jen Lai

**Affiliations:** 1 Institute of Medicine, Chung Shan Medical University, Taichung, Taiwan.; 2 NEVRON International Medical Center, Vladivostok, 690078, Russia; nevron.vl@gmail.com.; 3 Department of Medical Research, Chung Shan Medical University Hospital, Taichung, Taiwan.; 4 Department of Allergy, Immunology & Rheumatology, Chung Shan Medical University Hospital, Taichung, Taiwan.; 5 Department of Psychiatry, Chung Shan Medical University Hospital, Taichung, Taiwan.

**Keywords:** anti-inflammatory drugs, dementia, gout, old age.

## Abstract

**Introduction:** The interaction between hyperuricemia and the cognitive system is still under debate, with studies presenting somewhat conflicting results.

**Objectives:** This study aimed to investigate the risk of dementia in patients with gout who are administered anti-inflammatory drug treatment.

**Methods:** Gouty arthritis patients aged 50 years and older, who received at least one of the background therapy drugs (colchicine, corticosteroids, or nonsteroidal anti-inflammatory drugs for 6 months), were divided into the following groups and compared: patients who had dementia over a period of 5 years (n = 2,292) and matched patients without dementia (n = 2,292).

**Results:** We found that the most significant risk factors for dementia were stroke (OR, 2.66; 95% C.I., 2.33-3.03; AOR, 2.39; 95% C.I., 2.08-2.75) and depression (OR, 3.72; 95% C.I., 3.01-4.6; AOR, 3.25; 95% C.I., 2.60-4.05). The results of anti-gout drug administration, which impacted the dementia risk among patients of all ages (but especially in 50-64-year-old patients), demonstrated a higher risk ratio after 90 days of corticosteroid use (OR, 3.39; 95% C.I., 1.15-9.99), which was further increased after 180 days (OR, 3.61; 95% C.I., 1.31-9.94). We revealed that female patients experienced a significant increase in dementia risk after 90 days of corticosteroid administration, whereas male patients experienced a significant increase only after 180 days (OR, 1.52; 95% C.I., 1.06-2.17).

**Conclusion:** We had identified that > 90-day corticosteroid administration is a significant dementia risk factor in both female and male patients of all ages, especially in the 50-60-year-old group.

## Introduction

Hyperuricemia, which is traditionally considered a significant risk factor in the pathogenetic mechanism of gout, is responsible for the clinical manifestations of this disease: gouty arthritis, tophi, gouty nephropathy, and urinary stone disease 1. The vast majority of researchers rely on the 2016 updated European League Against Rheumatism (EULAR) recommendations for gout management 2, according to which the recommended uric acid level is > 360 μmol/l (6,8 mg/dl) irrespective of gender.

Gout is unusual in young people, but if onset occurs before 30 years of age, the condition is much more severe. Gout is often observed in multiple family members, and patients with metabolic syndrome are at risk of gout. According to expert opinion, approximately 0.1%-1% of the adult population suffers from gout, with a male to female ratio of 4:1. The peak incidence occurs at 40-50 years of age in men and ≥ 60 years of age in women 2-4.

The “partners” of hyperuricemia are “friends of affluence” 5. Hyperuricemia is associated with dyslipidemia, arterial hypertension, diabetes mellitus, insulin resistance, obesity, atherosclerosis, and other cardiovascular disorders 6. The National Health and Nutrition Examination Survey I study found an independent association between hyperuricemia and increased cardiovascular mortality. The 12-year study showed that the concentration of uric acid in blood serum is a predictor of cardiovascular prevalence and mortality. In addition, gout is characterized by typical changes in the regulation system of the blood aggregate state. Altogether, these factors indicate the systematicity and polymorbidity of gout.

Uric acid's pathophysiological role in the cognitive decline of patients with or without a specific diagnosis of dementia remains unknown 7-10. The interaction between hyperuricemia and cognitive function is still under debate, with studies presenting somewhat conflicting results 11-13.

The inverse association between serum uric acid levels and risk of dementia was confirmed by the Na Du meta-analysis in 2016. A total of 21 case-control studies of three cohort studies (total of 10 953 participants) were analyzed and showed that high serum uric acid levels were significantly associated with decreased risk of Alzheimer's disease 14.

The same observation in support of the neuroprotection of uric acid was presented by Lieke E. et al. In 1968-1969, they investigated a sample population of 1462 women, aged 38-60 years old, and observed them over a period of 44 years. It was noted that a higher level of sU (standard deviation 76,5 mcmole/l) was associated with a lower risk of dementia 15.

However, among other studies, Latourte A et al.'s 13 study of the elderly French population (≥ 65 years) showed an association between hyperuricemia, a higher risk of dementia, and MRI changes in the aging brain (extensive white mater hyperintensity volume), providing the first clinicopathological correlation to date between hyperuricemia and brain changes. A higher risk of dementia among gout patients should not be surprising, since gout is associated with hyperuricemia, chronic inflammation, and oxidative stress, and several or all of these mechanisms may play a key role in the pathogenesis of dementia 9.

The treatment of gout may appear to be an exhausted topic. Over the past 25 years, not a single fundamentally new anti-gout drug has been created. However, practice shows that not all issues in the treatment of gout have been resolved 16, 17. Gout flare-up medications include colchicine, nonsteroidal anti-inflammatory drugs (NSAIDs), and steroids, which can be taken together in severe cases and are most efficient when taken early after the onset of the flare-up 17.

Oral prednisolone, at a daily dose of 30-35 mg per day, has been shown to be effective and is recommended by the American College of Rheumatology (ACR) and League Against Rheumatism (EULAR) as a potential first-line therapy in the management of gout flare-ups 18, 19.

It is noteworthy that even a single dose of steroids can worsen hypertension and diabetes 20. In a Russian study 21, the frequency of side effects experienced following even a single intra-articular application of glucocorticosteroids (betamethasone 7 mg) was very high: the authors observed a clinically significant increase in systolic blood pressure in 73% of patients, fasting glycemia in 23% of patients, and myocardial ischemia in 13% of patients.

Long-term glucocorticosteroid therapy (prednisolone, 10 mg per day, for more than 3 months) can cause serious side effects, such as steroidogenic diabetes, arterial hypertension, atherosclerosis, thrombosis, hyperlipidemia, intestinal issues, renal impairment, immunodeficiency, and osteoporosis. Glucocorticosteroids also cause behavior and mood disorders, regardless of the patient's previous mental state. Cognitive side effects of steroids, related to attention and memory deficit (“glucocorticoid cascade hypothesis”), have not been adequately explored in the literature 22.

Hong J. et al. 10 found an association between gout and a reduced risk of dementia which was limited to treated gout patients (administered urate-lowering drugs or colchicine), and they observed no association among untreated gout patients, indicating that protection against dementia risk in gout patients may be medication-related. These observations raise the question of whether gout, independent of its treatment, increases the risk of dementia in the elderly 12.

In this study, based on controversial data from the literature, we attempt to determine the complex relationship between hyperuricemia and gout and identify risk factors for dementia, including age, gender, background factors, and medication side effects.

### Aim

We aimed to investigate the risk of dementia in gout patients receiving anti-inflammatory drug treatment.

## Materials and methods

### Data source

The National Health Insurance (NHI) program was implemented in Taiwan on March 1, 1995 and now covers 99.5% of the population. The recorded medical data were managed by the National Health Insurance Research Database (NHIRD). To react immediately and effectively to current situations, the National Health Research Institutes (NHRI) cooperated with the NHI Bureau (NHIB) for the creation and maintenance of the NHI Research Database. The NHRI protects the privacy of patients and transfers medical insurance data from the NHIB to health researchers seeking to improve the health of Taiwanese citizens. The data presented in this study were also derived from the NHIRD; access was approved by the NHRI Review Committee. The data consisted of inpatient and outpatient treatment records and registration files and were based on an academic, retrospective cohort group.

The International Classification of Diseases, 9th Revision, Clinical Modification (ICD-9-CM) Volumes 1 and 2 (including the Official ICD-9-CM Guidelines for Coding and Reporting), maintained and distributed by the Department of Health and Human Services, for coding diseases, injuries, impairments, other health problems and their manifestations, and causes of injury, disease, impairment, or other health problems, was used for the coding of the diseases 23. The Longitudinal Health Insurance Database (LHID) consisted of a random sample of 1 million individuals from the NHIRD, with data relating to the period from 1999 to 2013. The Ethics Committee of China Medical University and Hospital approved this study (CMUH-104-REC2-115). As information in NHIRD is initially codified, informed consent was waived by the ethics committee.

### Case and control selection

The study was a nested case-control study. Newly diagnosed gouty arthritis patients (≥ 50 years) who received an anti-inflammatory drug (ICD-9-CM = 274.0) from 2000 to 2008 were selected in the LHID. All patients received at least one of the background therapy drugs (colchicine (Anatomical Therapeutic Chemical (ATC) code: M04AC01), corticosteroids (ATC code: M01A), and NSAIDs (ATC code: M01A)) for 6 months. To ensure that only new-onset dementia cases were included, we excluded diagnoses made before the first gout diagnosis date. Participants in the case group were newly diagnosed with dementia (ICD-9-CM = 290.0-290.4, 294.1, 331.0-331.2), with at least two or more outpatient visits or one admission after a diagnosis of gout 5 years apart. The index date was the date of the first dementia diagnosis. The control group was never diagnosed with dementia after diagnosis of gout.

### Matching and confounders

We balanced the distribution of incidence of age, sex, and year of gout diagnosis between the two groups. A 1:1 matching system was used, allowing the control group to have the same index date. The comorbidities were hypertension (ICD-9-CM = 401-405), hyperlipidemia (ICD-9-CM = 272.0-272.4), chronic liver disease (ICD-9-CM = 571), chronic kidney disease (ICD-9-CM = 585), diabetes (ICD-9-CM = 250), chronic obstructive pulmonary disease (COPD) (ICD-9-CM = 491, 492, 496), autoimmune disease (ICD-9-CM = 710.0, 714.0, 720.0), cardiovascular disease (ICD-9-CM = 410-414), stroke (ICD-9-CM = 430-438), depression (ICD-9-CM = 296.2, 296.3, 300.4, 311), and Parkinson's disease (ICD-9-CM = 332), which were defined 5 years before the index date and after at least two or more outpatient visits or one hospitalization. The usage of warfarin (ATC code: B01AA03) and statin (ATC code: C10AA01, C10AA02, C10AA03, C10AA04, C10AA05, C10AA07, C10AA08) was included 5 years before the index date.

### Statistical analysis

To compare the demographic characteristics of the dementia and non-dementia groups, the chi-square test for categorical variables and Student's t-test for continuous variables were used. Conditional logistic regression was used in estimating the risk factors of dementia: this is a prolongation of logistic regression and allowed the consideration of stratification and matched data. The statistical software used was SPSS version 18.0 (SPSS Inc., Chicago, IL, USA).

## Results

**Figure 1:**
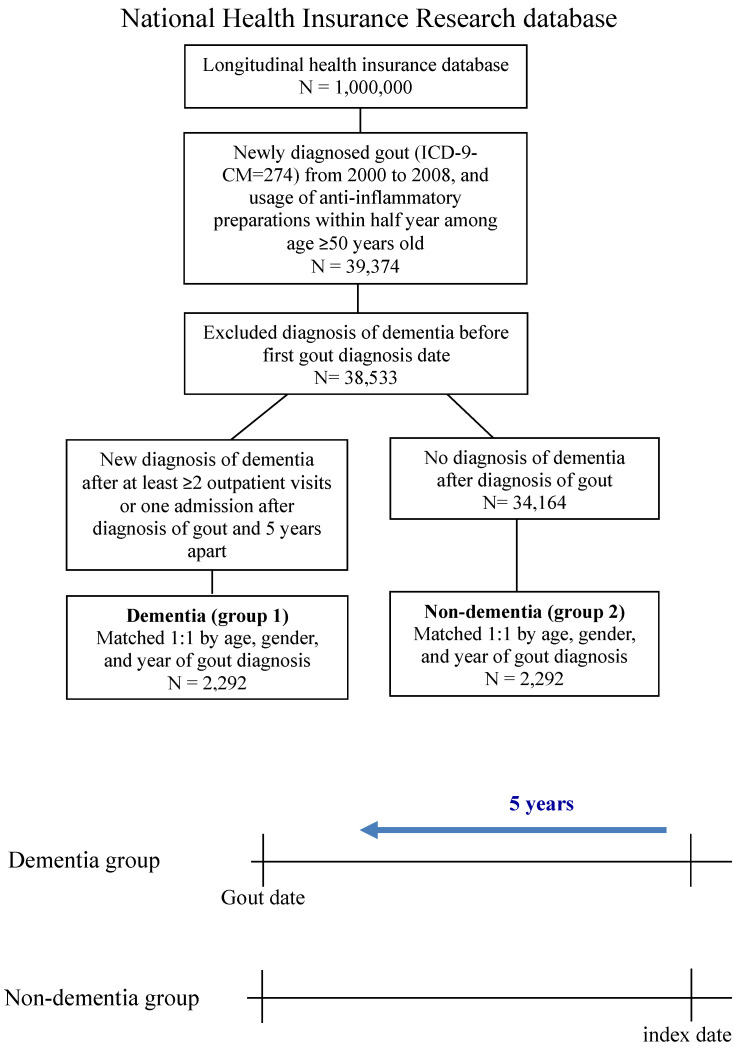
The flowchart of the process of obtaining a cohort of two groups: patients with gout and dementia and without dementia.

In our study population, 38,533 patients aged 50 years and older suffered from gout and used anti-inflammatory drugs during the period 2000-2008. After data processing, subjects were divided into two groups: “newly diagnosed with dementia” after confirmation of diagnosis of “gout” or development of dementia during the first 5 years (N = 2,751) and “never diagnosed with dementia” after diagnosis of gout (N = 34,164) (Fig. 1).

Table 1 shows the demographic characteristics of these two groups and epidemiological risk factors such as age, gender, and comorbidities.

Table 2 reveals the significant risk factors for dementia: drug administration (corticosteroids, warfarin, statin), hypertension, chronic liver disease, chronic kidney disease, COPD, cardiovascular disease, diabetes, and Parkinson's disease. However, the highest risk factors were stroke (OR: 2.66, 95% C.I.: 2.33-3.03; AOR: 2.39, 95% C.I.: 2.08-2.75), which increases dementia risk 2.66 times, and depression (OR, 3.72; 95% C.I., 3.01-4.6; AOR, 3.25; 95% C.I., 2.60-4.05), which increases dementia risk 3.72 times (Table 2). Other chronic diseases of the liver and kidneys, as well as diabetes, also influenced the development of dementia but carried approximately equal risk.

Results of anti-gout drug administration, which impacted the dementia risk in patients of all ages, demonstrated a higher risk ratio after 90 days of corticosteroid use, which increased further after 180 days (Table 3). In 50-64-year-old patients, the results also showed a significant increase in dementia risk in the surveyed cohort, by 3.39 times after 90 days of corticosteroid use (OR: 3.39, 95% C.I.: 1.15-9.99) and 3.61 times after 180 days (OR: 3.61, 95% C.I.: 1.31-9.94) (Table 4).

In > 65-year-old patients, we also found a 1.37-times significant increase in dementia risk after 90 days of corticosteroid administration and a 1.55-times increase after 180 days (Table 5).

We revealed that female patients experienced a 1.59-times significant increase in dementia risk after 90 days of corticosteroid administration and a 1.85-times increase after 180 days (Table 6).

Among male patients, a 1.52-times significant increase in dementia risk was found only after 180 days of corticosteroid administration (OR: 1.52; 95% C.I., 1.06-2.17) (Table 7).

## Discussion

In this study, we found an increased risk of dementia development among gout patients undergoing long-term (90 days or more) corticosteroid therapy, especially those aged between 50 and 64 years (OR: 3.6; 95% C.I., 1.31-9.94).

Women more often experience an increased risk in response to corticosteroids only, but men also experience increased risks following long-term therapy (more than 3 months) with NSAIDs (OR: 1.85; 95% C.I., 0.96-3.55). Gout patients are expected to take steroids for long periods, often continuously, because gout flare-ups become more frequent and last longer over time. However, this can significantly increase the risks of adverse effects, such as emotional, behavioral, and cognitive disorders, as well as dementia.

There are two classic approaches to the treatment of gout: colchicine or NSAID administration. At present, it is agreed that the efficacy of the two approaches is generally similar. The differences lie in the timing of the onset of the therapeutic effects and tolerance only. Colchicine takes effect more rapidly, after 12 to 48 hours, whereas NSAIDs take effect after 24 to 48 hours; however, these treatments also frequently involve side effects 24-26.

In cases where the use of NSAIDs or colchicine is impossible, glucocorticosteroids are prescribed. Two alternative methods to prevent gout flare-ups are known: intravenous colchicine and glucocorticosteroid (intra-articular, per os, or parenteral) or adrenocorticotrophic hormone treatment. It should be noted that the use of glucocorticosteroids (intravenous or intramuscular) in gout patients can cause a rebound effect and other side effects that may demand the hospitalization of the patient; hence, the simultaneous administration of small doses (1-2 mg/per day) of colchicine should be considered 27.

Some very interesting conclusions were presented at the annual EULAR Congress 2018 in Amsterdam. In a cohort that consisted of 1.71 million participants in the Medicare program, 111,656 new-onset dementia cases were reported. The total rate of incidence of dementia among subjects with or without gout was 10.9 and 17.9 per 100 man-years, respectively. In the multiparameter adjusted analysis, gout was independently related to a significantly higher risk ratio of new-onset dementia, with an OR of 1.15 (95% C.I., 1.12-1.18, analysis of sensitivity confirmed main data). Patients aged between 65 and 75 years, 75 and 85 years, and ≥ 85 years were associated with 3.5- and 7.8-times higher risks of the development of dementia, respectively; the risk was also higher for women, patients of African descent, and those with several comorbid diseases. Gout was independently related to a 15% higher risk of new-onset dementia in the elderly 9.

Chronic high levels of glucocorticosteroids can materially affect the functioning of the nervous system both positively and negatively. Some of the effects are initially manageable, but long-term effects can cause irreversible changes 27.

Researchers have paid careful attention to the mechanisms by which corticosteroids affect neuronal activity. It is important to note that the excitability of neurons can depend on the local production of steroids, called neurosteroids, in the brain 28. One of the most well-described pathologic changes in the nervous system is the atrophy of hippocampal neurons among male mice and rats, which shows a connection between high basal cortisol levels and hippocampus atrophy and memory problems in humans 29, 30.

Hippocampal atrophy, induced by corticosteroids, can play an important role in the pathogenesis of a wide spectrum of neuropsychiatric disorders. The hippocampus is needed for the consolidation of short-term memory and regulation of the hypothalamic-pituitary-adrenal axis 31. Signs of hippocampal damage (dysregulation of hypothalamic-pituitary-adrenal axis with memory problems) are found in patients with affective disorders, Alzheimer's disease (AD), and posttraumatic disorders. MRI volumetry showed reduced hippocampus size in patients with all the above mentioned disorders. Several questions remain unanswered regarding the chronology of neurodegeneration, causation, reversibility, types of damage, factors of hippocampal damage rate, and also pharmacological (corticotropin-releasing hormone antagonists, antiglucocorticoid drugs, GABA-ergic, serotonergic, and glutamatergic agents), and nonpharmacological (psychotherapy) treatment approaches 32.

Gout patients occasionally suffer from comorbidities that are incompatible with NSAID, colchicine, or cortisone treatment 16. As represented in our study, patients with gout in both the dementia and non-dementia groups demonstrated a very high frequency of comorbid pathologies before treatment: hypertension (83.8% and 78.9%), diabetes (41.5% and 34.1%), cardiovascular diseases (42.1% and 36.5%), and others.

Singh JA et al. 9 explained the possibility that the association between hyperuricemia and cardiovascular disease is due to concomitant oxidative stress (a hypothesis that remains to be proven), which is implicated in the pathogenesis of dementia, or due to its association with other cardiovascular disease risk factors. Interestingly, we determined comorbid cardiovascular disease in gout patients as a significant risk factor for dementia, which should be further examined in other elderly cohorts 18, 19.

In our study, we identified that the prolonged administration (> 90 days) of corticosteroids was associated with dementia risk in both female and male patients of all surveyed ages but especially in the 50-64-year-old group. Our results are comparable with Dregan A. et al.'s 33 data. The authors investigated 31,083 patients with AD; 23,465 with vascular dementia (VaD); and 1,694 with Lewy body dementia. Dregan A. et al.'s results showed that glucocorticoid (GC) drug administration was associated with a higher risk of VaD.

In 1984, Varney et al. highlighted the possibility that reversible dementia can occur during therapy with GCs without psychotic manifestations 34. On the other hand, the presence of psychotic manifestations during therapy with GCs must be carefully evaluated for a relationship with possible concomitant dementia 35-36. Wolkowitz et al. 37 published a case series of patients with significant cognitive impairment during GC therapy. In these patients, this impairment persisted after GC discontinuation as a consequence of steroid neurotoxicity. They proposed the term “steroid dementia syndrome” as the paradigm of a nonreversible state of dementia. The relationship between dementia and GCs must take into account also the disease for which GCs are used—for example, gout 34. High-dose corticosteroids (0.5-1.5 g prednisone per day) are the initial treatment in most patients, which may serve as a diagnostic test when the diagnosis is uncertain. Complete (or near-complete) resolution of cognitive impairment after high-dose GC treatment is very demonstrative 34.

However, the pathogenic mechanism behind corticosteroids' influence on the development of cognitive disorders including dementia is yet to be completely understood and requires further research.

### Limitations

In summary, our study presents the possibility to test the hypothesis of the effects of long-term anti-inflammatory drug (corticosteroids, in particular) therapy on the risk of dementia in gout patients. However, some limitations should be acknowledged.

First of all, NHIRD does not include data on the family history of dementia, social and economic statuses of patients, and their unhealthy habits and addictions. Also, there is no data on uric acid levels in the NHIRD database, so we can not compare uric acid levels between the two groups during the study.

Secondly, the NHIRD registers patients receiving anti-inflammatory therapy only, without consideration of combined therapy, disease severity, duration, and stage of disease, so it was impossible to assess these factors.

Thirdly, the variability in clinical manifestations of cognitive disorders (mild forms) and its diagnostics at the early stage when amnestic disorders are still absent can be difficult for clinicians to account for and may even be ignored by them. Alternatively, long-lasting pain syndrome, sleep disorder, invalidation of the patient, and depression can cause a “false-positive result” of dementia. In this case, the coefficient of the risk of dementia may prove to be biased if incorrect classification leads to statistical analysis changes. However, a typical feature of a case-control design study is that it does not identify a cause-and-effect link.

## Conclusion

In our investigation, we had identified that > 90-day corticosteroid administration is a significant dementia risk indicator in both female and male patients of all surveyed ages but especially in the 50-64-year-old group. Future research should aim towards the clarification of the role of steroids in dementia pathogenesis.

## Figures and Tables

**Table 1:** Demographic characteristics

	Dementia (N =2,292)		Non-dementia(N =2,292)		
	n	%	n	%	p-value
**Age (years)**					1
50-64	164	7.2	164	7.2	
65-79	1,320	57.6	1,320	57.6	
≥80	808	35.3	808	35.3	
Mean ± SD	76.7 ± 7.2		76.7 ± 7.2		1
**Gender**					1
Female	1,128	49.2	1,128	49.2	
Male	1,164	50.8	1,164	50.8	
Hypertension	1,920	83.8	1,809	78.9	<0.001
Hyperlipidemia	981	42.8	962	42.0	0.570
Chronic liver disease	497	21.7	432	18.8	0.017
Chronic kidney disease	277	12.1	208	9.1	0.001
Diabetes	951	41.5	781	34.1	<0.001
COPD	724	31.6	604	26.4	<0.001
Autoimmune disease	107	4.7	89	3.9	0.189
Cardiovascular disease	966	42.1	837	36.5	<0.001
Stroke	1,023	44.6	515	22.5	<0.001
Depression	429	18.7	135	5.9	<0.001
Parkinson's disease	262	11.4	59	2.6	<0.001
**Colchicine**	803	35.0	829	36.2	0.423
**Corticosteroids**	2,186	95.4	2,144	93.5	0.007
**Non-steroidal anti-inflammatory drugs**	2,260	98.6	2,257	98.5	0.712
Warfarin	116	5.1	73	3.2	0.001
Statin	872	38.0	793	34.6	0.015
**Gout year**					1
2000	712	31.1	712	31.1	
2001	463	20.2	463	20.2	
2002	356	15.5	356	15.5	
2003	253	11.0	253	11.0	
2004	187	8.2	187	8.2	
2005	135	5.9	135	5.9	
2006	97	4.2	97	4.2	
2007	54	2.4	54	2.4	
2008	35	1.5	35	1.5	
Study period (years)	7.8 ± 2.4		7.8 ± 2.3		0.907

COPD: Chronic obstructive pulmonary disease.

**Table 2:** Conditional logistic regression of risk of dementia

	Crude OR	95% C.I.	p-value	Adjusted OR^†^	95% C.I.	p-value
Colchicine	0.95	0.84-1.07	0.410	0.96	0.84-1.11	0.597
Corticosteroids	1.43	1.11-1.86	0.007	1.06	0.79-1.41	0.700
NSAIDs	1.09	0.68-1.77	0.714	0.90	0.54-1.52	0.701
Hypertension	1.40	1.2-1.63	0.000	1.01	0.85-1.2	0.937
Hyperlipidemia	1.04	0.92-1.17	0.564	0.82	0.7-0.97	0.020
Chronic liver disease	1.19	1.03-1.37	0.018	1.12	0.96-1.32	0.158
Chronic kidney disease	1.40	1.15-1.7	0.001	1.20	0.97-1.49	0.096
Diabetes	1.37	1.22-1.55	0.000	1.32	1.15-1.51	0.000
COPD	1.30	1.14-1.48	0.000	1.14	0.99-1.32	0.075
Autoimmune disease	1.21	0.91-1.6	0.195	1.22	0.89-1.67	0.218
Cardiovascular disease	1.27	1.13-1.43	0.000	1.07	0.94-1.23	0.301
Stroke	2.66	2.33-3.03	0.000	2.39	2.08-2.75	0.000
Depression	3.72	3.01-4.6	0.000	3.25	2.60-4.05	0.000
Warfarin	1.62	1.2-2.19	0.002	1.29	0.92-1.80	0.140
Statin	1.17	1.03-1.32	0.014	1.11	0.94-1.31	0.224

COPD: Chronic obstructive pulmonary disease. †Adjusted for colchicine, corticosteroids, NSAIDs, hypertension, hyperlipidemia, chronic liver disease, chronic kidney disease, diabetes, COPD, autoimmune disease, cardiovascular disease, stroke, depression, warfarin, and statin.

**Table 3:** Conditional logistic regression of risk of dementia (Drugs, period of treatment)

	N	No. of dementia	Crude OR	95% C.I.	p-value	Adjusted OR^†^	95% C.I.	p-value
Colchicine (days)								
None	2,952	1,489	1			1		
<90	1,009	493	0.94	0.81-1.08	0.383	0.95	0.8-1.11	0.497
90-179	192	98	1.02	0.76-1.36	0.906	1.24	0.89-1.72	0.197
≥180	431	212	0.95	0.77-1.17	0.615	0.98	0.77-1.24	0.859
Corticosteroids (days)								
None	254	106	1			1		
<90	2,249	1,084	1.30	1-1.7	0.051	0.97	0.72-1.3	0.834
90-179	804	412	1.47	1.11-1.97	0.008	1.05	0.76-1.46	0.760
≥180	1,277	690	1.65	1.25-2.17	0.000	1.06	0.77-1.46	0.707
NSAIDs (days)								
None	67	32	1			1		
<90	1,286	575	0.88	0.54-1.43	0.606	0.81	0.47-1.39	0.444
90-179	870	457	1.20	0.73-1.97	0.471	1.01	0.59-1.75	0.961
≥180	2361	1228	1.18	0.73-1.91	0.502	0.88	0.51-1.5	0.636

†Adjusted for colchicine, corticosteroids, NSAIDs, hypertension, hyperlipidemia, chronic liver disease, chronic kidney disease, diabetes, COPD, autoimmune disease, cardiovascular disease, stroke, depression, warfarin, and statin.

**Table 4:** Conditional logistic regression of risk of dementia (Age = 50-64)

	N	No. of dementia	Crude OR	95% C.I.	p-value	Adjusted OR^†^	95% C.I.	p-value
Colchicine (days)								
None	215	109	1			1		
<90	76	36	0.87	0.5-1.52	0.624	0.80	0.35-1.86	0.612
90-179	11	6	1.12	0.33-3.84	0.859	1.04	0.17-6.57	0.964
≥180	26	13	0.96	0.4-2.3	0.928	0.53	0.15-1.86	0.322
Corticosteroids (days)								
None	25	8	1			1		
<90	193	87	1.52	0.59-3.89	0.384	0.65	0.19-2.31	0.510
90-179	48	30	3.39	1.15-9.99	0.027	1.77	0.41-7.56	0.441
≥180	62	39	3.61	1.31-9.94	0.013	2.20	0.53-9.08	0.277
NSAIDs (days)								
None	4	2	1			1		
<90	125	46	0.64	0.08-4.85	0.664	0.71	0.06-8.82	0.793
90-179	80	47	1.70	0.21-13.67	0.616	2.62	0.2-35.04	0.467
≥180	119	69	1.57	0.21-11.91	0.664	1.74	0.14-21.18	0.662

†Adjusted for colchicine, corticosteroids, NSAIDs, hypertension, hyperlipidemia, chronic liver disease, chronic kidney disease, diabetes, COPD, autoimmune disease, cardiovascular disease, stroke, depression, warfarin, and statin.

**Table 5:** Conditional logistic regression of risk of dementia (Age ≥65)

	N	No. of dementia	Crude OR	95% C.I.	p-value	Adjusted OR^†^	95% C.I.	p-value
Colchicine (days)								
None	2737	1380	1			1		
<90	933	457	0.94	0.81-1.1	0.446	0.95	0.8-1.12	0.543
90-179	181	92	1.01	0.75-1.36	0.940	1.25	0.89-1.74	0.195
≥180	405	199	0.95	0.76-1.17	0.621	0.98	0.77-1.26	0.896
Corticosteroids (days)								
None	229	98	1			1		
<90	2056	997	1.27	0.96-1.67	0.097	0.94	0.69-1.28	0.673
90-179	756	382	1.37	1.02-1.86	0.039	0.97	0.69-1.37	0.882
≥180	1215	651	1.55	1.16-2.07	0.003	0.99	0.71-1.39	0.970
NSAIDs (days)								
None	63	30	1			1		
<90	1161	529	0.92	0.55-1.52	0.731	0.85	0.49-1.48	0.573
90-179	790	410	1.17	0.71-1.96	0.537	1.01	0.58-1.77	0.971
≥180	2242	1159	1.17	0.71-1.93	0.537	0.89	0.51-1.55	0.681

†Adjusted for colchicine, corticosteroids, NSAIDs, hypertension, hyperlipidemia, chronic liver disease, chronic kidney disease, diabetes, COPD, autoimmune disease, cardiovascular disease, stroke, depression, warfarin, and statin.

**Table 6:** Conditional logistic regression of risk of dementia (Female)

	N	No. of dementia	Crude OR	95% C.I.	p-value	Adjusted OR^†^	95% C.I.	p-value
Colchicine (days)								
None	1647	839	1			1		
<90	406	190	0.84	0.67-1.05	0.130	0.84	0.66-1.08	0.172
90-179	72	36	0.95	0.6-1.52	0.834	0.99	0.6-1.66	0.983
≥180	131	63	0.89	0.63-1.28	0.539	0.91	0.6-1.36	0.635
Corticosteroids (days)								
None	105	42	1			1		
<90	1141	545	1.37	0.91-2.07	0.135	1.12	0.71-1.78	0.615
90-179	446	230	1.59	1.03-2.45	0.037	1.25	0.77-2.03	0.373
≥180	564	311	1.85	1.21-2.84	0.005	1.40	0.86-2.27	0.179
NSAIDs (days)								
None	26	16	1			1		
<90	531	229	0.47	0.21-1.06	0.070	0.48	0.2-1.15	0.100
90-179	416	210	0.63	0.28-1.41	0.259	0.59	0.24-1.42	0.237
≥180	1283	673	0.69	0.31-1.54	0.368	0.58	0.24-1.4	0.228

†Adjusted for colchicine, corticosteroids, NSAIDs, hypertension, hyperlipidemia, chronic liver disease, chronic kidney disease, diabetes, COPD, autoimmune disease, cardiovascular disease, stroke, depression, warfarin, and statin.

**Table 7:** Conditional logistic regression of risk of dementia (Male)

	N	No. of dementia	Crude OR	95% C.I.	p-value	Adjusted OR^†^	95% C.I.	p-value
Colchicine (days)								
None	1305	650	1			1		
<90	603	303	1.02	0.84-1.23	0.851	1.05	0.84-1.31	0.656
90-179	120	62	1.08	0.74-1.56	0.698	1.50	0.97-2.31	0.067
≥180	300	149	0.99	0.77-1.29	0.961	1.09	0.81-1.48	0.559
Corticosteroids (days)								
None	149	64	1			1		
<90	1108	539	1.26	0.89-1.79	0.187	0.85	0.57-1.26	0.412
90-179	358	182	1.39	0.94-2.06	0.098	0.90	0.57-1.42	0.645
≥180	713	379	1.52	1.06-2.17	0.023	0.85	0.55-1.31	0.460
NSAIDs (days)								
None	41	16	1			1		
<90	755	346	1.30	0.69-2.47	0.418	1.11	0.55-2.25	0.775
90-179	454	247	1.85	0.96-3.55	0.064	1.39	0.67-2.87	0.379
≥180	1078	555	1.63	0.87-3.07	0.130	1.06	0.52-2.15	0.881

†Adjusted for colchicine, corticosteroids, NSAIDs, hypertension, hyperlipidemia, chronic liver disease, chronic kidney disease, diabetes, COPD, autoimmune disease, cardiovascular disease, stroke, depression, warfarin, and statin.
